# The relationship between childhood trauma and adulthood anxiety and depression among Tunisian university students

**DOI:** 10.1192/j.eurpsy.2024.1719

**Published:** 2024-08-27

**Authors:** M. Turki, W. Hammemi, S. Ellouze, M. Barkallah, I. Mannoubi, N. Halouani, J. Aloulou

**Affiliations:** ^1^Psychiatry “B” department, Hedi Chaker university hospital; ^2^Higher college of health science, Sfax, Tunisia

## Abstract

**Introduction:**

Traumatic childhood has increasingly high incidence rates and can be predictive of negative health outcomes. There is a large consensus indicating that childhood trauma is significantly involved in the development of mood disturbances in adulthood.

**Objectives:**

The aim of this study was to examine the relationship between retrospectively recalled childhood trauma and adulthood anxiety and depression in a sample of undergraduate university students.

**Methods:**

A cross-sectional study was conducted among a sample of 365 university students randomly selected from 8 universities in Sfax (Tunisia). Information about childhood maltreatment, depressive and anxiety symptoms were gathered through the Childhood Trauma Questionnaire-Short Form (CTQ-SF), and the Hospital Anxiety and Depression Scale (HADS) respectively. To test the hypothesis, examining the relationship between anxiety, depression, and childhood trauma, we used Spearman’s correlation test. Multivariate logistic regression models were used as well.

**Results:**

The mean age of our participants was 20.3 years. More females (68.2%) than males participated in the study. Our findings showed that the five childhood trauma subtypes (emotional abuse and neglect, physical abuse and neglect, sexual abuse) were significantly correlated with anxiety and depression symptoms severity (p<0.01). Emotional abuse was the strongest risk factor for adulthood anxiety symptoms (OR=6.002, 95% CI= [3.238; 11.125]) while emotional neglect was the strongest risk factor for depressive symptoms in adulthood (OR=6.214, 95% CI= [3.428; 11.267]). Multivariate analysis revealed that, in subjects with childhood trauma, scores of anxiety symptoms were positively and highly associated with the severity of emotional abuse (adjusted B=1.438, 95% CI= [1.951; 9.092], p=0.000). Depression symptoms severity were as well positively correlated with severity of emotional abuse (adjusted B=0.848, 95% CI= [1.043; 5.224], p=0.039), and severity of emotional neglect (adjusted B=1.044, 95% CI= [1.263; 6.389], p=0.012).

**Conclusions:**

This study highlighted the relevance of childhood trauma as a factor contributing to anxiety and depression in adulthood. Thus, early psychological support of victims of childhood trauma can reduce the rate of anxiety and depression among these subjects.

**Disclosure of Interest:**

None Declared

